# Circular RNA ame_circ_2015 Function as microRNA Sponges in Regulating Egg-Laying of Honeybees (*Apis mellifera*)

**DOI:** 10.3390/life13010161

**Published:** 2023-01-05

**Authors:** Xiao Chen, Deqian Wang, Jiandong An

**Affiliations:** 1 Institute of Apicultural Research, Chinese Academy of Agricultural Sciences, Beijing 100193, China; 2 Institute of Animal Husbandry and Veterinary Science, Zhejiang Academy of Agricultural Sciences, Hangzhou 310021, China

**Keywords:** honeybees, the number of egg-laid, circRNA, miRNA

## Abstract

Honeybees (*Apis mellifera*) are critical to maintaining ecological balance and are important pollinators. The oviposition behavior in honeybees is important and complex. Circular RNAs (circRNAs) are found to form circRNA-miRNA crosstalk and play important roles in reproduction processes. Here, dual luciferase reporter was used to confirm the crosstalk between ame_circ_2015 and ame_miR-14-3p. Functional experiments in vitro and in vivo were performed to investigate the biological functions of ame_circ_2015 in egg-laying of queens. The results showed that ame_circ_2015 directly target ame_miR-14-3p, and the expression of ame_circ_2015 was negatively correlated with ame_miR-14-3p expression. Overexpression results showed that ame_circ_2015 promoted the number of eggs laid and knockdown of ame_circ_2015 suppressed the number of eggs laid. It demonstrates that up-regulated ame_circ_2015 promotes the number of eggs laid by sponging ame_miR-14-3p. The study will provide information towards a better understanding of circRNA-miRNA crosstalk in egg-laying in honeybees.

## 1. Introduction

Honeybees (*Apis mellifera*) are important pollinators and are critical to ecology balance [1]. As one of the important traits in honeybees, oviposition behavior is complex. In honeybees, queens specialize in oviposition. In the developing season, queens lay approximately 1500 eggs per day under ideal circumstances. In winter, queens stop laying eggs for several months. They constantly adjusted the number of eggs laid according to nutrition, season and so on. The number of eggs laid are closely related to the development of the bee colony [2]. High number of eggs laid promotes the rapid reproduction and development of the colony, and further improves the beekeeping economic benefits. Therefore, we focus on the number of eggs laid of queens.

Circular RNA (circRNA) is a new type of non-coding RNAs [3,4]. CircRNA is more stable than linear RNA, so it is more ubiquitous in life [5]. It can competitively bind miRNAs through sequence binding sites, thereby releasing miRNAs’ inhibitory effect on its target genes, and further effect the expression of target genes [4]. Therefore, circRNAs’ roles in regulation of biological processes cannot be ignored, and it has become a new hot spot in the study of RNA regulation of biological processes, including reproduction processes [6,7,8]. Researchers have identified a large number of circRNAs in humans, mice, fruit flies, pigs, cattle, sheep and other species. It is found that circRNAs are especially involved in regulation of the reproduction processes [6,7,8]. For example, in 2014, Westholm et al. identified circRNA molecules in *Drosophila* ovarian tissues, and found that their expression showed tissue specificity [9]; in 2015, Fan et al. found that circRNAs were abundantly expressed in pre-implantation embryos and play important roles in embryonic development [10]; in 2017, Tao et al. identified circRNAs in goat ovarian tissue and found that circRNAs might affect the development of goat ovarian follicles by target miR-34, miR-483 and miR-1468 [11]. Our previous study found that circRNAs were enriched in queens’ ovaries and participated in the ovarian activation and the initiation of egg-laying [12]. However, the effect of circRNAs on the number of eggs laid of queens is still unclear. Based on the previous work, this study further studied the effect of circRNAs on the eggs-laid number in queens and the possible regulation’s mechanism of egg-laying.

In this study, we focus on one circRNA, ame_circ_0002015 (simplified as circ_2015, sequence information showed in Table S1) [12]. Circ_2015 was up-regulated significantly (*p* < 0.05) in the ovary activation process [12]. It has binding sites of ame_miR-14-3p (simplified as miR-14-3p), and they can form circ_2015-miR-14-3p crosstalk [12]. MiR-14-3p was reported to affect the eggs-laid number by targeting EcR [13]. EcR is important in egg-laying in honeybees for its role in the ecdysteroids pathway. Ec (used hereafter to refer to all types ecdysteroids) and its receptor are indispensable for successful egg-laying [14,15,16]. So, circ_2015 may also interact with miR-14-3p and further affect Ec pathway, and participate in regulating egg-laying. However, it was not clear how the expression of circ_2015 and miR-14-3p were related during honeybees’ egg-laying and whether the expression of circ_2015 was directly associated with the number of eggs laid by the queens. In this study, we used luciferase assay to confirm the crosstalk between circ_2015 and miR-14-3p. The knocking-down and overexpression of circ_2015 in the queen model were implemented to confirm the effect of circ_2015 expression on the number of eggs laid. The study will provide information towards a better understanding of circRNA-miRNA crosstalk in egg-laying in honeybees.

## 2. Materials and Methods

### 2.1. Sampling

The honeybee colonies used in the study were maintained by Institute of Apicultural Research, Chinese Academy of Agricultural Sciences (IAR, CAAS), Beijing, China. More than 100 *Apis mellifera ligustica* virgins with the same genetic source were used in the study. After sexual maturity, each virgin was artificially inseminated with the same volume of mixed sperm (8 μL) (QueenBee Artificial Insemination Instrument VCFE-QBAII-H1.3, Victory & Explore Ltd., Shanghai, China). The mixed sperm were collected from 1000 drones from three different queens’ families. After successfully laying eggs, each queen was introduced to its own colony. Each colony has the similar size. The number of eggs laid by each queen was checked and around 800 eggs per day were observed. All queens were included in the next siRNA injection experiment.

Each queen was injected with 1 μL siRNA solution (2.5 μg/μL, 5 μg/μL and 7.5 μg/μL, respectively) or 1 μL nonsense sequence. The procedure of injection was implemented according to Amdam’s methods [17]. In specific, the queen was injected dorsally between the 5th and 6th abdominal segment. In an hour after injection, the queen should stay in the fixed position to avoid the solution backflow. Then, the queen was moved back to the original colony. The number of eggs laid was observed the next day and recorded for three days. After that, queens were collected and samples of ovaries were taken. Each sample consisted of the ovary from one queen and was used for the further RNA extraction (total = 90 samples).

### 2.2. RNA Extraction and Quantitative Real Time PCR

Total RNA was extracted using Trizol reagent (Invitrogen, Carlsbad, CA, USA) according to the manufacturer’s instructions. The purity, concentration and integrity of RNA were assessed, and all qualified RNA was used in next experimental protocol.

For first strand cDNA synthesis of circRNA, reverse transcription reaction was performed using M-MLV FIRST STRAND KIT (Invitrogen, Shanghai, China) and oligo (dT)^18^ primer. Then, transcript quantification was performed (Table 1). The RT-qPCR reactions were prepared using SYBR Green mix (Roche Diagnostics GmbH, Roche Applied Science, Mannheim, Germany). An amount of 5 μL of cDNA (50 ng, 1:100 dilution) was included in a total volume of 20 μL. β-actin served as the internal reference gene.

For first strand cDNA synthesis of miRNA, reverse transcription reaction was performed using miRcute Plus miRNA First-strand cDNA Kit (TIANGEN, Beijing, China). Then, transcript quantification was performed using miRcute Plus miRNA qPCR Kit (SYBR Green). An amount of 5 μL of cDNA (50 ng, 1:100 dilution) was included in a total volume of 20 μL. U6 served as the internal reference gene.

By standard curve calculation, PCR efficiency of each gene was estimated. Ct values were transformed to quantities and relative gene expression was calculated using the 2^−ΔΔCt^ method.

### 2.3. S2 Cell Culture and Luciferase Reporter Assay

To test whether miR-14-3p actually targets circ_2015, a luciferase assay was performed using a dual-specific luciferase assay kit (Biyuntian, Shanghai, China). Two luciferase reporter plasmids were designated, named as psiCHECK2-circ_2015 and psiCHECK2-circ_2015-Mut. For psiCHECK2-circ_2015, we sub-cloned the complete sequence of circ_2015 (1142-bp) including the predicted miR-14-3p recognition site. For psiCHECK2- circ_2015-Mut, the predicted miR-14-3p recognition site was changed to mutations. For cell culture, *Drosophila* S2 cells were used in the study. Cultured with 24 h, miR-14-3p agomir/antagomir was co-transfected with either psiCHECK2-circ_2015, psiCHECK2- circ_2015-Mut, or an blank vector as control in the cells using the method as described by Tiscornia et al. [18]. Twenty-four hours and 48 h after transfection, the firefly luciferase activity of each sample was examined and was normalized to the renilla luciferase activity.

### 2.4. Overexpression and Inhibition of circ_2015

To overexpress circ_2015, an overexpressed vector was constructed. The complete sequence of circ_2015 was cloned into a pcDNA3.1 vector and amplified using PCRMasterMix (SinoGene, Beijing, China) (Table S1). To inhibit the expression of circ_2015, a siRNA sequence and a nonsense control were synthesized (GenePharma, Shanghai, China) (Table 2). Injection of siRNA and queens’ sampling were implemented as described in Sampling part.

### 2.5. Statistical Analysis

A one-way ANOVA and a t-test were conducted to analyze the RNA expression in different groups. Data were analyzed by software SPSS 22.0 [19].

## 3. Results

### 3.1. Confirmation of the Interaction between circ_2015 and miR-14-3p

**Figure 1:**
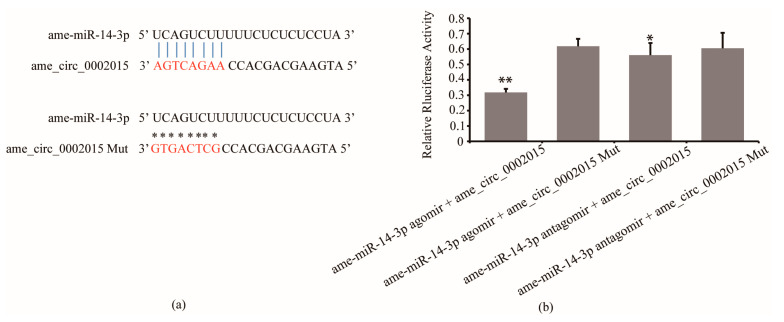
Interaction between circ_2015 and miR-14-3p. (**a**) The target sites between miR-14-3p and circ_2015. Nucleotides of target sites and mutated sites of circ_2015 are shown in red. The vertical blue lines indicate contiguous Watson-Crick pairing and asterisks indicate no pairing with mutated sites. (**b**) Confirmation of the interaction between circ_2015 and miR-14-3p using a luciferase reporter assay. A normalized renilla luciferase value was plotted with mean ± SD. miR-14-3p agomir + circ_2015, miR-14-3p agomir co-transfected with psiCHECK2-circ_2015; miR-14-3p agomir + circ_2015 Mut, miR-14-3p agomir co-transfected with psiCHECK2-circ_2015 Mut; miR-14-3p antagomir + circ_2015, miR-14-3p antagomir co-transfected with psiCHECK2-circ_2015; miR-14-3p antagomir + circ_2015 Mut, miR-14-3p antagomir co-transfected with psiCHECK2-circ_2015 Mut. * *p* < 0.05, ** *p* < 0.01.

The results of luciferase reporter assay confirmed the interaction between circ_2015 and miR-14-3p. It showed that the luciferase activity significantly decreased in the group of miR-14-3p agomir co-transfected with psiCHECK2-circ_2015 compared to the group of miR-14-3p agomir co-transfected with psiCHECK2-circ_2015 Mut (*p* < 0.01, Figure 1b). And the luciferase activity significantly decreased in the group miR-14-3p antagomir co-transfected with psiCHECK2-circ_2015 compared to the group of miR-14-3p antagomir co-transfected psiCHECK2-circ_2015 Mut (*p* < 0.05, Figure 1b). The results indicated that miR-14-3p targeted circ_2015.

### 3.2. The Regulation Mechanism of miR-14-3p on circ_2015

**Figure 2:**
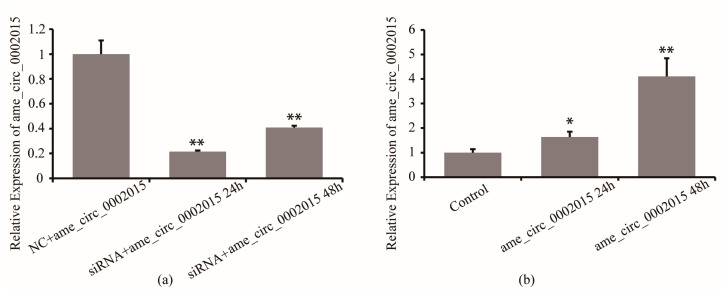
Overexpression and inhibition of circ_2015. (**a**) Effect of siRNA on the expression of circ_2015. (**b**) Effect of overexpressed vector on the expression of circ_2015. NC + circ_2015, nonsense sequence co-transfected with psiCHECK2-circ_2015; siRNA + circ_2015 24 h, circ_2015 siRNA co-transfected with psiCHECK2-circ_2015 at 24 h; siRNA + circ_2015 48 h, circ_2015 siRNA co-transfected with psiCHECK2-circ_2015 at 48 h; Control, vector; circ_2015 24 h, psiCHECK2-circ_2015 transfected at 24 h; circ_2015 48 h, psiCHECK2-circ_2015 transfected at 48 h. * *p* < 0.05, ** *p* < 0.01.

Firstly, the efficiency of circ_2015 siRNA in S2 cells was detected. Twenty-four hours and 48 h after circ_2015 siRNA and NC co-transfected into S2 cells, the expression of circ_2015 was detected. The results showed that the circ_2015 expression decreased by 80% at 24 h and decreased by 60% at 48 h (*p* < 0.01). The results showed that the inhibition caused by siRNA was effective (Figure 2a). Then, the efficiency of psiCHECK2-circ_2015 was examined. PsiCHECK2-circ_2015 and a blank vector were co-transfected into S2 cells, respectively. Twenty-four and 48 h after transfection, the expression of circ_2015 was detected. The results showed that the expression of circ_2015 increased significantly at 24 h (*p* < 0.05), and was increased around 3.1 times at 48 h (*p* < 0.01, Figure 2b).

**Figure 3:**
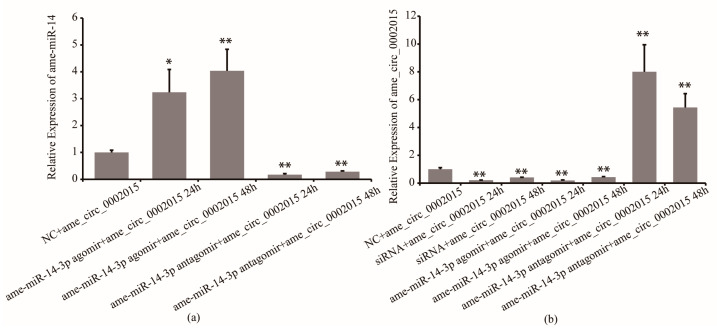
The regulation mechanism of miR-14-3p on circ_2015. (**a**) The expression of miR-14-3p after miR-14-3p agomir/antagomir transfection. (**b**) The expression of circ_2015 after miR-14-3p agomir/antagomir transfection. NC + circ_2015, nonsense sequence co-transfected with psiCHECK2-circ_2015; miR-14-3p agomir + circ_2015 24 h, 24 h after miR-14-3p agomir co-transfected with psiCHECK2-circ_2015; miR-14-3p agomir + circ_2015 48 h, 48 h after miR-14-3p agomir co-transfected with psiCHECK2-circ_2015; miR-14-3p antagomir + circ_2015 24 h, 24 h after miR-14-3p antagomir co-transfected with psiCHECK2-circ_2015; miR-14-3p antagomir + circ_2015 48 h, 48 h after miR-14-3p antagomir co-transfected with psiCHECK2-circ_2015; siRNA + circ_2015 24 h, 24 h after circ_2015 siRNA co-transfected with psiCHECK2-circ_2015; siRNA + circ_2015 48 h, 48 h after circ_2015 siRNA co-transfected with psiCHECK2-circ_2015. * *p* < 0.05, ** *p* < 0.01.

Furthermore, the regulation effect of miR-14-3p on circ_2015 was confirmed. Twenty-four hours and 48 h after miR-14-3p agomir/antagomir and psiCHECK2-circ_2015 co-transfected, the expression of circ_2015 was detected. It showed that in the miR-14-3p inhibition group, the expression of miR-14-3p decreased by 83% at 24 h (*p* < 0.01) and decreased by 72% at 48 h (*p* < 0.01), respectively (Figure 3a). In the miR-14-3p overexpressed group, the expression of miR-14-3p increased by 223% at 24 h (*p* < 0.05) and increased by 300% at 48 h (*p* < 0.01), respectively (Figure 3a). For the expression of circ_2015, in the miR-14-3p inhibition group, the expression of circ_2015 increased almost 7 times at 24 h (*p* < 0.01) and increased around 4.4 times at 48 h (*p* < 0.01), respectively (Figure 3b). In the miR-14-3p overexpressed group, the expression of circ_2015 decreased by 81% at 24 h (*p* < 0.01) and decreased by 57% at 48 h (*p* < 0.01), respectively (Figure 3b). The results showed that miR-14-3p affects the expression of circ_2015. It indicated that miR-14-3p may inhibit the transcription of circ_2015.

### 3.3. Circ_2015 Expression and Its Effect on the Number of Egg-Laid of Queens

**Figure 4:**
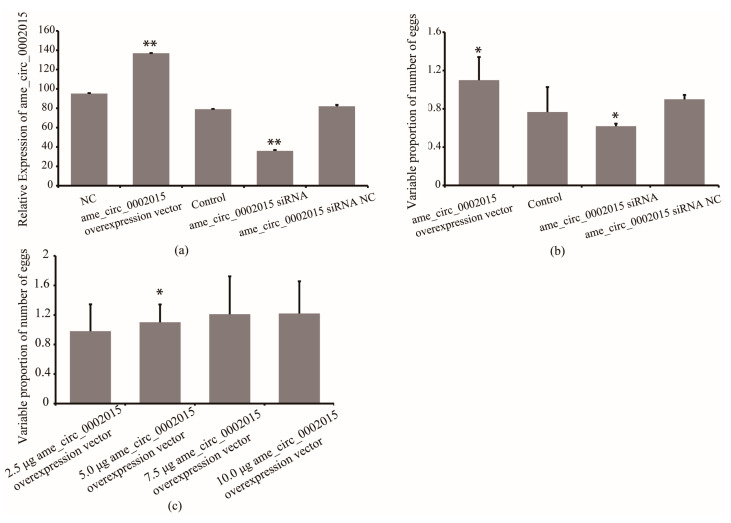
Effect of circ_2015 on the number of egg-laid of queens. (**a**) The expression of circ_2015 in queens after treatment. (**b**) The effect of circ_2015 on the number of egg-laid of queens. (**c**) The circ_2015 titer-dependent effect on the number of egg-laid of queens. NC, queens without treatment group. circ_2015 overexpression vector, circ_2015 overexpression group; control, blank vector group; circ_2015 siRNA, circ_2015 siRNA group; circ_2015 siRNA NC, nonsense sequence control group; 2.5 μg circ_2015 overexpression vector, overexpression group with 2.5 μg circ_2015; 5.0 μg circ_2015 overexpression vector, overexpression group with 5.0 μg circ_2015; 7.5 μg circ_2015 overexpression vector, overexpression group with 7.5 μg circ_2015; 10.0 μg circ_2015 overexpression vector, overexpression group with 10.0 μg circ_2015. * *p* < 0.05, ** *p* < 0.01.

To further confirm whether circ_2015 affects the number of eggs laid by queens, the expression of circ_2015 was overexpressed and inhibited, and its association with egg-laid number was analyzed. The results showed that the expression of circ_2015 increased by 73% in the overexpressed group compared with that of the control group (*p* < 0.01). The expression of circ_2015 decreased by 56% the siRNA group compared with that of the siRNA NC group (*p* < 0.01, Figure 4a).

The RT-qPCR confirmed the overexpression and inhibition of circ_2015 in queens, respectively. As shown in Figure 4, the circ_2015 expression in queens from the overexpressed group increased by 73% compared with that of the control group (*p* < 0.01), while circ_2015 expression from the siRNA group decreased by 56% compared with that of the siRNA NC group (*p* < 0.01, Figure 4a). The association results showed that the number of egg-laid increased by 43% in the circ_2015 overexpressed group compared with the control (*p* < 0.05) (Figure 4b). The eggs-laid number decreased by 31% in the circ_2015 siRNA group compared with the NC group (Figure 4b). The results indicated that the expression of circ_2015 has an effect on the number of eggs laid.

To further confirm the circ_2015 titer-dependent effect on the number of eggs laid, four groups with different amount of synthetic reagent were implemented (Figure 4c). The result showed that the number of eggs laid increased significantly between the 2.5 μg group and the 5.0 μg group (*p* < 0.05), but not significantly between the 5.0 μg and the 7.5 μg group. The number of eggs laid did not increase between the 7.5 μg group and the 10.0 μg group.

## 4. Discussion

Honeybees are an indispensable species for maintaining the sustainable development of the ecological environment. One of the important traits of honeybee queens is egg-laying. Excellent queens with a high eggs-laid number can rapidly develop the colony size. This will make a good preparation for the further pollination and production. However, the genetic architecture of reproductive traits is complex. Additionally, the heritability is low. The eggs-laid number cannot be measured until queens’ sexual maturity and this makes early selection impossible. By using traditional selection methods, the genetic improvement would be slow. Fortunately, with the development of molecular biology, there are new methods to help improve complex traits, such as molecular marker-assisted selection and genome selection [20]. Using these methods, remarkable genetic improvement have been achieved in domestic animals. Therefore, research on the identification of molecular markers in honeybees was also performed.

CircRNAs play an important role in various biological processes and regulate genes’ expression. With the development of molecular biology, more studies on circRNAs’ roles in reproduction processes were reported. For example, Song et al. found that the expression of circRNAs was related with estrogen(E2)/progesterone(P4) and affect the endometrium receptiveness during embryo implantation in goats [21]. Zhang et al. found that the circRNAs affect gonadotropin-releasing hormone activities and further influence the reproduction of sheep [22,23]. Studies on animals have shown that circRNAs play important roles in reproductive traits. In honeybees, studies on circRNAs in egg-laying is not reported. Our previous study found that circRNAs were enriched in queens’ ovaries and participated in the ovarian activation and egg-laying [12]. However, the effect of circRNAs on the number of eggs laid in queens is still unclear. Based on the previous work, this research further studied the effect of circRNA_2015 on the number of eggs laid by queens and the possible mechanism of regulating egg-laying.

To determine whether the expression of circ_2015 was directly associated with the eggs-laid number of queens, we recorded the number of eggs laid per day by each queen and analyzed the association between the circ_2015 expression and the number of eggs laid by queens. The results indicate that the overexpression of circ_2015 promotes the number of egg-laid and the inhibition of circ_2015 repress the number of egg-laid. Thus, we deduce that there is a good correlation between the expression of circ_2015 and the number of eggs laid of queens. Next, we detected whether the circ_2015 titers was related with the number of eggs laid. The results showed that accompany with the increasing of circ_2015 titer, the number of eggs laid increased too. In our previous study, we also reported that circ_2015 was only found in queens’ ovaries instead of workers [12]. The results indicated that the expression of circ_2015 affects the number of eggs laid.

The possible mechanism of circ_2015 affecting the number of egg-laid in queens was analyzed in two directions. Firstly, we analyzed the parental gene of circ_2015, which is zinc finger protein ush (LOC100577801). Multitype zinc-finger proteins of the Friend of GATA/U-shaped (Ush) regulated genes’ expression by their modulation of GATA factor activity. In *drosophila*, zinc finger protein ush is dynamically expressed during embryonic processes. It was reported that the Ush transcriptional regulator played a critical role in embryogenesis in *drosophila* [24]. The genomic homology of *Apis mellifera* with *Drosophila melanogaster* is high, so each genes’ function may be similar too. Our previous study also found that zinc finger protein ush was up-regulated in ovarian activation process [25]. Additionally, functional annotation results showed that zinc finger protein ush was involved in ectoderm development and larval organs development. Recent studies reported that circRNAs have a similar function with their parental genes [26]. Therefore, we deduced that circ_2015 also play a role in egg genesis. Combining the results of the association between circ_2015 expression and the number of egg-laid, we deduced that circ_2015 may participate in egg genesis and further affect the egg-laid number.

The second possible mechanism of circ_2015 affecting oviposition is circRNA-miRNA crosstalk. CircRNAs can competitively bind miRNAs through sequence-binding sites, thereby releasing miRNA’s inhibitory effect on its target gene, and finally regulating the target genes’ expression [27,28]. In human, circ_000839 as the target of miR-200b inhibited the expression of RhoA and further played roles in tumorigensis [29]. We previously predicted that circ_2015 sponged miR-14-3p by bioinformatics analysis [12]. Our luciferase assay result confirmed that circ_2015 sponged miR-14-3p and the overexpression of circ_2015 significantly inhibited the expression of miR-14-3p. The results strongly indicated they form the circ_2015-miR-14-3p crosstalk and further regulate biological processes. The roles of miRNAs in reproduction of honeybees have been well established [30,31,32]. Specially, miR-14-3p is found to play roles in regulating the activity of EcR and further affect the queens’ ovarian state [31,33]. We also found that EcR had a binding site of miR-14-3p, and they form EcR-miR-14-3p crosstalk [13]. Through this crosstalk, miR-14-3p negatively regulated EcR’s expression and activity [13]. EcR is important for its roles in regulating Ec titer, and further regulates egg-laying [14,15,16]. Additionally, we previously found that miR-14-3p affected the number of eggs laid by targeting EcR [13]. Inhibition of miR-14-3p expression enhanced the number of eggs laid by queens [13]. So, in summary, the overexpression of circ_2015 decreased the miR-14-3p expression, and the decreased expression of miR-14-3p further promotes the expression of EcR. Additionally, the high expression of EcR influences the Ec pathway, and finally affects the egg-laying of queens. Combining results of the association between the number of eggs laid and gene abundance, we deduce that circ_2015 might affect the number of eggs laid by sponging miR-14-3p and further regulating the expression of target gene EcR [14,34,35,36].

With the development of molecular biology technology and the discovery of a large number of biological genetic markers, the genetic and breeding of honeybees has been further developed. In recent years, studies showed that a complex interaction network formed by coding and non-coding RNA affect the egg-laying of honeybees [12,13,30,32,37,38]. The joining of circRNAs makes the network even more complex. However, circRNAs also helps us to understand the regulation mechanism of egg-laying more deeply. This study found that the overexpression of circ_2015 increases the number of eggs laid of queens. CircRNAs, as molecular markers, can be used in genetic improvement in honeybees combining with traditional selection methods. However, other genes or loci may interact with circ_2015. Further studies are needed to investigate the specific mechanisms.

## 5. Conclusions

In summary, we detected that circ_2015 sponged miR-14-3p, and the overexpression of circ_2015 enhanced the number of eggs laid of queens. These findings suggested that circ_2015, by sponging miR-14-3p, played an important role in regulating egg-laying of honeybee queens.

## Figures and Tables

**Table 1:** Primer sequences used for RT-qPCR.

Primer	5′ to 3′
circ_2015-F	CGGAGTTAGACTATAGGAGTC
circ_2015-R	CTCCGCGTAGCGTGGAAGAGT
β-actin-F	CTGCTGCATCATCCTCAAGC
β-actin-R	GAAAAGAGCCTCGGGACAAC
miR-14-3p-F	GCGCTCAGTCTTTTTCTCT
U6	CTTGCTTCGGCAGAACATAT

**Table 2:** Primers for cloning, overexpression and inhibition of circ_2015.

Gene Name	Primer or siRNA
circ_2015 sub-cloning	F: 5′-AATTCTAGGCGATCGCTCGAGGGGAGGATGAGGAATGGA-3′ R: 5′-ATTTTATTGCGGCCAGCGGCCGCCTCGAATTTGTTCGACTTCTC-3′
circ_2015 overexpressed vector constructing	F: 5′-TTTATACTTCAGGATGGGGAGGATGAGGAATGGA-3′ R: 5′-ACCGGTATCGATGATCTCGAATTTGTTCGACTTCTC-3′
circ_2015 siRNA	sense: 5′-GAACAAAUUCGAGGGGGAGGAU-3′ antisense: 5′-UCCUCCCCCUCUAAUUUGUUCTT-3′
siRNA nosense control	sense: 5′-UUCUCCGAACGUGUCACGUTT-3′ antisense: 5′-ACGUGACACGUUCGGAGAATT-3′

## Data Availability

Not applicable.

## Supplementary Materials

The following supporting information can be downloaded at: https://www.mdpi.com/article/10.3390/life13010161/s1, Table S1: Sequence information of ame-circ_0002015.
