# Supplementary material for: Circular RNA ame_circ_2015 Function as microRNA Sponges in Regulating Egg-Laying of Honeybees (Apis mellifera)
Source: Life (Basel). 2023 Jan 5;13(1):161. doi: 10.3390/life13010161 (PMC9865145; doi:10.3390/life13010161)
Supplement: Supplementary file 1 [file life-13-00161-s001.zip › life-2086084-supplementary.pdf]

Table S1 Sequence information of ame_circ_2015

Type	information
Name	ame_circ_0002015
Chromosome	LG11
Parental gene	*Apis mellifera* zinc finger protein ush (*LOC100577801*)
Circled exons	Circled by three exons of *LOC100577801* (170779..171035, 171250..171646, 171910..172647)
Junction site	GACGAGGAGAAGTCGAACAAATTCGAGGGGGAGGATGAGGA ATGGAGCGACTCG
miRNA target site	AAGACTGA
Sequence	5’GGGGAGGATGAGGAATGGAGCGACTCGGAGAAGACACCTT CGGTGAGCAGTGGAGTGGAGGGCGGTGGATCGGCGGTCTCG AGCCCGATCGCGCCGATCGTCGAAGAGGAATCGAGTTTGCCG CCACCGCCGAGACTGAACCCCCCTTCCTCGTCGGTTGTGATCG CGAATTCGGCCGCTGAGGATATCAGATTGAGGCTCAGCGTTCT CTCTGAAGATGCTCGGAAACGGCTGGCTAGTCTCACCGAGGA TATCGAGGGTCGCGAGAAGTTTGAGAGACCGACATGCAGCGC CAAGGGCCAATCAAACCCGAGAATCGAGCCCGAAGGACACG GACGAGGATTCCAACATGGTGGTGGTCAAGGAAGAGGATTGC CAGCCGAGGTCGTCGTCCTCCTCGTCTTCCTCGACCAGAAGG CGGGAGTCCGTGTGCAGGAGGGACTCGGAGGACTCCTCGAG ACGATCGAACGCGGACGAGACACCGTCGGAGAAAAAGCTGA AACTCGATGATGAAGCAGCACCAAGACTGAGACTCAATGCTA GTCTGGCGACGGATCCCGCGCTTCGGCCCGCCGCTGTAGCCG CGCTCACCGTCAAACCGGAAAATACCTCGCCGCCGAATCCTG TGCCACCCCTGCCGGCTGGACTTCAGAACGGGTAACTATATTG CAATCATTAGATCGAATAGAGATTTCTTCTAGACGTATTTATGC CGGGAAAAAATGCTAGAAAGAAGGGAAAGATAGATTAGATCG GTAATTCTGTCGTTAAATCGCAATAGTTTCATTCTTTCCAATCC AACAATTTCAAAGATCAACGTGGTTCCAAACGATATATCATCG TCCTATCTGCTCGCGTATCATACATAAAACTGCATGCAAAAGTA TACGTAACTGAGTGGAAGCGGACCAACGTTGCAGGCGATCGC GTCGGGTCGGCTGTTCGTGCTGCCAACCGACGGCAAGGAGAC GATCACGGTGGAGCCAGCCAGGCCGGCGCCCCTCATCTGCCC GCCATGCGGCATCCGCTTTAGCTCGGCGAGCACCCTCGAGGC GCATCGCACCTTCTACTGCGCCCATCGTCCTCGGCTCGAGGAG GAGGCGGCCAACGAGGAGGACGAGGAGAAGTCGAACAAATT CGAG3’
